# Gene Expression Array Analyses Predict Proto-Oncogene Expression During Perineural Invasion in Pancreatic Ductal Adenocarcinoma

**DOI:** 10.5152/tjg.2024.21430

**Published:** 2024-01-01

**Authors:** Shu Zhao, Zhen Xue, Jing-Yao Wang, Peng Song

**Affiliations:** 1 Department of Oncology, The Second Medical Center and National Clinical Research Center for Geriatric Diseases, Chinese PLA General Hospital, Beijing, China; 2 Department of Oncology, Tianjin Medical University General Hospital, Tianjin, China; 3 Department of Imaging, Beijing Mentougou District Hosptal, Beijing, China

**Keywords:** Perineural invasion, pancreatic ductal adenocarcinoma, differentially expressed genes, bioinformatics analysis

## Abstract

**Background/Aims:**

Pancreatic ductal adenocarcinoma is the tumor type with the highest incidence of perineural invasion. This study tries to identify the differentially expressed genes regulated between pancreatic ductal adenocarcinoma tissues with perineural invasion and without perineural invasion.

**Materials and Methods:**

The GSE102238 profile was downloaded. Gene function and pathway analysis were subsequently conducted. A protein–protein interaction network was constructed to search for hub genes. Both univariate Cox analysis and multivariate Cox analysis were calculated to identify prognostic factors. Quantitative real-time polymerase chain reaction (RT-PCR) and overall survival analysis of hub genes were used to verify.

**Results:**

Our study identified 242 differentially expressed genes including 68 upregulated differentially expressed genes and 174 downregulated differentially expressed genes, which were involved in important functions and pathways. Nine relevant core genes using protein–protein interaction analysis as well as nestin (NES)/vascular endothlial growth factor (VEGF) signaling pathway which is highly related to the pathological process of perineural invasion in pancreatic ductal adenocarcinoma were also discovered. The differentiation was identified as an independent prognostic factor (*P < .*05) after multivariate Cox analysis. Three upregulated genes (*JUP*,* CALM1*, and *NES*) and 6 downregulated genes (*EPHA2*,* ARF1*, *ORM2*,* TERT*,* IL18*, and *CXCL3*) were validated by quantitative RT-PCR and they all had markedly worse overall survival (*P < .*05).

**Conclusion:**

This analysis showed that 9 core genes including *JUP*,* CALM1*,* NES*,* EPHA2*,* ARF1*,* ORM2*,* TERT*,* IL18*, and* CXCL3*, as well as NES/VEGF signaling pathway, have a relationship with the development process of perineural invasion in pancreatic ductal adenocarcinoma. Cox analysis and overall survival analysis suggested differentiation as an independent prognostic factor and key roles for these 9 hub genes in perineural invasion prognosis in pancreatic ductal adenocarcinoma.

**Main Points**

- Two hundred forty-two differentially expressed genes (DEGs) were identified including 68 upregulated DEGs and 174 downregulated DEGs screened in pancreatic ductal adenocarcinoma (PDAC) tissue from patients diagnosed with perineural invasion (PNI).
- Nine relevant core genes, including *JUP*,* CALM1*,* NES*,* EPHA2*, *ARF1*,* ORM2*,* TERT*,* IL18*, and* CXCL3*, as well as NES/VEGF signaling pathway, have a relationship with the development process of PNI in PDAC.
- The differentiation is an independent prognostic factor and key roles for these 9 prognostic genes in PNI prognosis in PDAC were validated.

## Introduction

Pancreatic cancer (PC) has become one of the most threatening tumors to human beings because of its difficult early diagnosis, strong invasion, low resection rate, high mortality, and short survival time. Although PC accounts for only 1%-3% of all malignancies, the incidence rate has increased year by year in recent years, and only 15%-20% of patients can finally undergo radical surgery.^1-3^ Even though the basic and clinical research on PC has made progress in recent years, the prognosis of PC is still grim, as well as the overall survival (OS) rate of 5 years is less than 5%.^4^ Studies have shown that PC has a high incidence of perineural invasion (PNI), even up to 100%. This may be a way of metastasis of PC and an important cause of the recurrence of PC. Perineural invasion-positive patients are always associated with poor prognosis and low survival rate.^5,6^

Perineural invasion means that cancer cells invade the adventitia and perineurium and even reach the neurointima and Schwann cells and neurons closely associated with it.^7^ Pancreatic ductal adenocarcinoma (PDAC) is the tumor type with the highest incidence of PNI, but the tumor size is not necessarily related to the occurrence of PNI, even if cancer can be seen only under the microscope, PNI can still occur. The occurrence of PNI is related to the neurophilicity of PC cells and the close anatomical location of the pancreas and nerve plexus. The distribution of the nerve plexus makes good contact between cancer cells and nerves. Cancer cells can directly invade the nerve and can also invade the nerve through penetrating channels (such as blood vessels and reticular fibers).^8-12^ Perineural invasion has been considered as an extension of lymphatic metastasis for many years because of the presence of lymphatic vessels in the adventitia. In recent years, it was found that the lymphatic vessels did not penetrate the epineurium, so the relationship between PNI and lymphatic metastasis was excluded.^13,14^

The molecular mechanism of PNI in PC is extremely complex, and most studies have focused on the interaction between nerve and tumor cells, and few have paid attention to the changes in the tumor matrix and microenvironment. The invasion of PC cells into peripheral nerves not only provides a pathway for the metastasis of PC but also leads to neural remodeling and changes in the neural environment, thus profoundly affecting the microenvironment of PC.^15,16^ Through the expression profile GSE102238 from the Gene Expression Omnibus database (GEO) and bioinformatics analysis, this study was undertaken to elucidate the differentially expressed genes (DEGs) between 50 pairs of PDAC tissues and matched non-tumor tissues, of which 28 pairs were diagnosed as PNI by experienced pathologists, to explore the downstream molecules associated with PNI gene. By doing this, we hope to provide a novel understanding of the molecular basis of the effect of PNI on the microenvironment of PC.

## Materials and Methods

The study was approved by the ethics committee of the Chinese PLA General Hospital (approval no. 2021PS213).

### Gene Expression Microarray Data

The Gene expression profile GSE102238 was downloaded from the GEO (GEO, www.ncbi.nlm.nih.gov/geo/). GSE102238 was based on Agilent-052909 CBC_lncRNAmRNA_V3 platform. The GSE102238 dataset contained PDAC tissue from patients diagnosed with PNI (n = 28) and PDAC tissue from patients without PNI (n = 22).

### Differentially Expressed Genes in Pancreatic Ductal Adenocarcinoma Tissue

**Figure 1.**
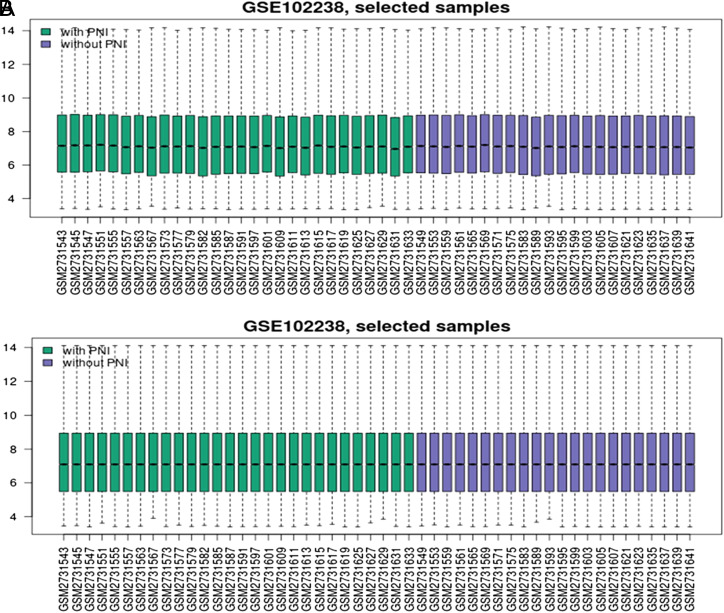
The box plots of the selected sample gene expression data before (A) and after (B) normalization in the GSE102238 dataset. Green bars: pancreatic ductal adenocarcinoma (PDAC) tissue samples from patients diagnosed with perineural invasion (PNI); purple bars: PDAC tissue samples from patients diagnosed without PNI.

Raw data were TXT files, which were assessed with GEO2R, which compares original submitter-processed data tables utilizing the GEO query and limma R packages (Bioconductor software). The volcano maps and box plots were completed using ggplot2 package R software and GEO2R online tools to illustrate the differential appearance. The box plots of the selected sample gene expression data before and after normalization are shown in Figure 1. Log Fold Change (FC) >0.5 or log FC <−0.5 as well as *P < .*05 were cutoffs to determine DEGs between PDAC tissue samples from patients diagnosed with PNI and PDAC tissue specimens from patients without PNI.

### Gene Ontology and Kyoto Encyclopedia of Genes and Genome Analyses

Cytoscape v3.4.0 (www.cytoscape.org) and ClueGO v2.33 were applied for identifying Gene Ontology (GO) and pathway analysis. Gene Ontology analysis including biological process (BP), molecular function (MF) and cellular component (CC), and Kyoto Encyclopedia of Genes and Genome (KEGG) analysis could determine the distinguishable genes’ expression patterns and roles among these differentially expressed mRNAs. Overrepresented pathways showing *P < .*05 were deemed statistically significant.

### Gene Interaction Network Generation

Multiple DEGs in this work might be PNI associated and could be involved in PNI progression in the PDAC microenvironment. First, DEGs were entered in the Search Tool for the Retrieval of Interacting Genes (STRING) database (http://www.string-db.org/), which yielded an interaction network (combined score above 0.4). Next, protein–protein interaction (PPI) networks comprising PNI-related genes of humans were obtained with Cytoscape v3.4.0. Core gene distribution in this network was generated with NetworkAnalyzer. Then, the Molecular Complex Detection (MCODE) plugin of Cytoscape was used for screening the network’s modules.

### Cox Regression Analysis

Univariate Cox analysis of OS with the “survival” package in R was conducted to identify prognostic factors including clinical features with significant prognostic value, and *P < .*05 was considered to be statistically significant. The independent prognostic factors were identified by multivariate Cox regression analysis with the “survival” package in R. The clinical data were gathered from the GEO database and utilized to screen for the independent prognostic factor. The variables including age, gender, sizes, tumor node metastasis classification (TNM) stages, differentiation, localization of tumor, vessel invasion, and hub genes were included. *P*-values in univariate Cox analysis were calculated with log-rank method. If the previously mentioned variables with *P < .*05 in univariate Cox analysis, they would be included in multivariate Cox analysis, and the variables with *P < .*05 in multivariate Cox analysis could be regarded as statistically significance. Consequently, the differentiation was identified as an independent prognostic factor (*P < .*05).

### Quantitative Real-Time Polymerase Chain Reaction

Total RNA was extracted by using Trizol Reagent (Ambion, Austin, TX, USA) from the 3 pairs of PDAC tissues with and without PNI of 6 patients with pathologically confirmed PDAC. The cDNA synthesis was performed using Reverse Transcription Kit (VAZYME, Nanjing, China). Quantitative RT-PCR was then carried out with the RT-PCR Kit (VAZYME). Following the manufacturer’s instructions, the thermal program underwent 10 minutes at 95°C, then 40 cycles of amplifications, 15 seconds at 95°C for denaturation, 60 seconds at 60°C for annealing, and 15 seconds at 95°C for the extension. The RT-PCR were carried out on QuantStudio 6 Thermal Cycler (ABI, Houston, TX, USA) using SYBR Green PCR Master Mix (VAZYME). An internal control including GAPDH, and the sequence of all the primers used have been described in Supplementary Table 1. Each sample was analyzed and calculated in triplicate. The 2^−ΔΔCt^ method was used to calculate the relative quantification of the hub genes.

### Survival Analysis

In the OS assessment, 50 patients were assigned to the low and high groups based on the median expression levels of various hub genes. Then, Kaplan–Meier survival curves were generated with the “survival” package in R to compare the differences reported in the OS.

### Statistical Analysis

Statistical analysis was performed based on R software (version 4.1.0). The chi-square test or Fisher’s exact test was used to analyze the categorical variables. The *t*-test and one-way analysis of variance (ANOVA) were used to analyze the continuous variables. Univariate and multivariate Cox regression and log-rank tests were performed to evaluate OS. Unless otherwise stated, *P < .*05 indicated that the difference was statistically significant.

## Results

### Differentially Expressed Genes in Pancreatic Ductal Adenocarcinoma Tissue

**Figure 2.**
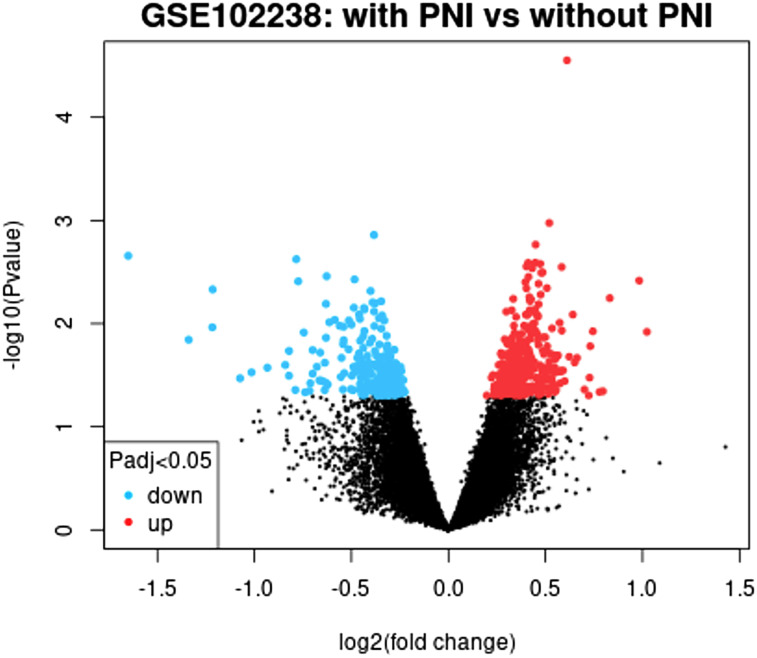
Volcano plot of all differentially expressed genes (DEGs) between pancreatic ductal adenocarcinoma (PDAC) tissue samples from patients diagnosed with perineural invasion (PNI) and without PNI in the GSE102238 dataset. Red dots: significantly upregulated genes; blue dots: significantly downregulated genes; black dots: non-differentially expressed genes. *P < .*05 was considered statistically significant.

We downloaded the gene profile GSE102238 from the GEO and used GEO2R algorithm to confirm DEGs in PDAC tissue from patients diagnosed with PNI compared with PDAC tissue from patients without PNI, which were shown in the volcano plot (Figure 2). Using the cutoff criteria, 242 DEGs including 68 upregulated DEGs and 174 downregulated DEGs screened in PDAC tissue from patients diagnosed with PNI compared with PDAC tissue from patients without PNI were discovered based on the whole expression profile, top 10 DEGs were listed in Table 1 and a complete differential gene expression table was included as a supplementary file (Supplementary Table 2).

### Gene Ontology Annotation Analysis of Differentially Expressed Genes

**Figure 3.**
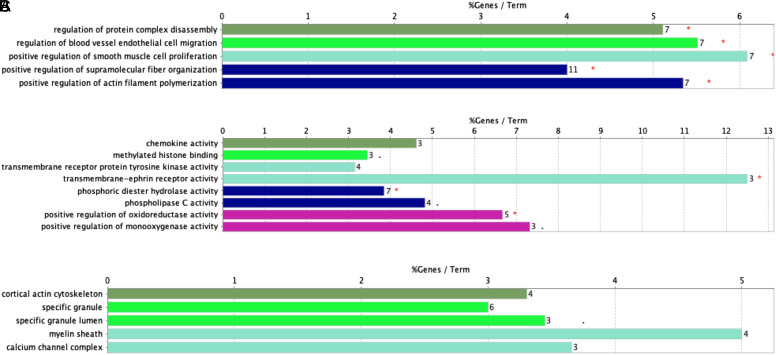
Gene Ontology (GO)-enrichment analysis of biological processes (A), molecular functions (B), and cellular components (C). The red star in GO terms means Term *P* < .05, and double red stars in GO terms mean Term *P* < .01.

Functional analysis of the 242 DEGs was revealed using the Cytoscape software. Target genes were annotated to the GO pathway, which significantly enriched in the regulation of protein complex disassembly, regulation of blood vessel endothelial cell migration, positive regulation of smooth muscle cell proliferation, positive regulation of supramolecular fiber organization, positive regulation of actin filament polymerization and other biological processes (Figure 3). For MF, the DEGs were enriched in chemokine activity, methylated histone binding, transmembrane receptor protein tyrosine kinase activity, transmembrane-ephrin receptor activity, phosphoric diester hydrolase activity, phospholipase C activity, positive regulation of oxidoreductase activity, positive regulation of monooxygenase activity, and others. In addition, GO CC analysis also showed that the DEGs were significantly enriched in the cortical actin cytoskeleton, specific granule, specific granule lumen, myelin sheath, calcium channel complex, and others.

### Kyoto Encyclopedia of Genes and Genome Enrichment Analysis of Differentially Expressed Genes

Target genes were annotated to the KEGG pathway, which enriched in NF-κB signaling pathway, biosynthesis of the N-glycan precursor (dolichol lipid-linked oligosaccharide, LLO) and transfer to a nascent protein, uptake and actions of bacterial toxins, stimuli-sensing channels, RAB GEFs exchange GTP for GDP on RABs, Rab regulation of trafficking, signaling by VEGF, VEGFA-VEGFR2 pathway, VEGFR2-mediated vascular permeability, signaling by ERBB2, PI3K/AKT signaling in cancer, downregulation of ERBB2 signaling, opioid signaling, G-protein-mediated events, PLC beta-mediated events, EPH-ephrin signaling, EPHA-mediated growth cone collapse, EPH-ephrin-mediated repulsion of cells, and others. Table 2 showed all core pathways and corresponding genes summarized. The first-ranking VEGFA-VEGFR2 pathway and downregulation of ERBB2 signaling had the 10.34% associated genes, which included *AKT3*,* CALM1*,* JUP*,* MAPKAPK3*,* NCF2*, and *RASA1*.

### Interaction Network of Differentially Expressed Genes and Core Genes

**Figure 4.**
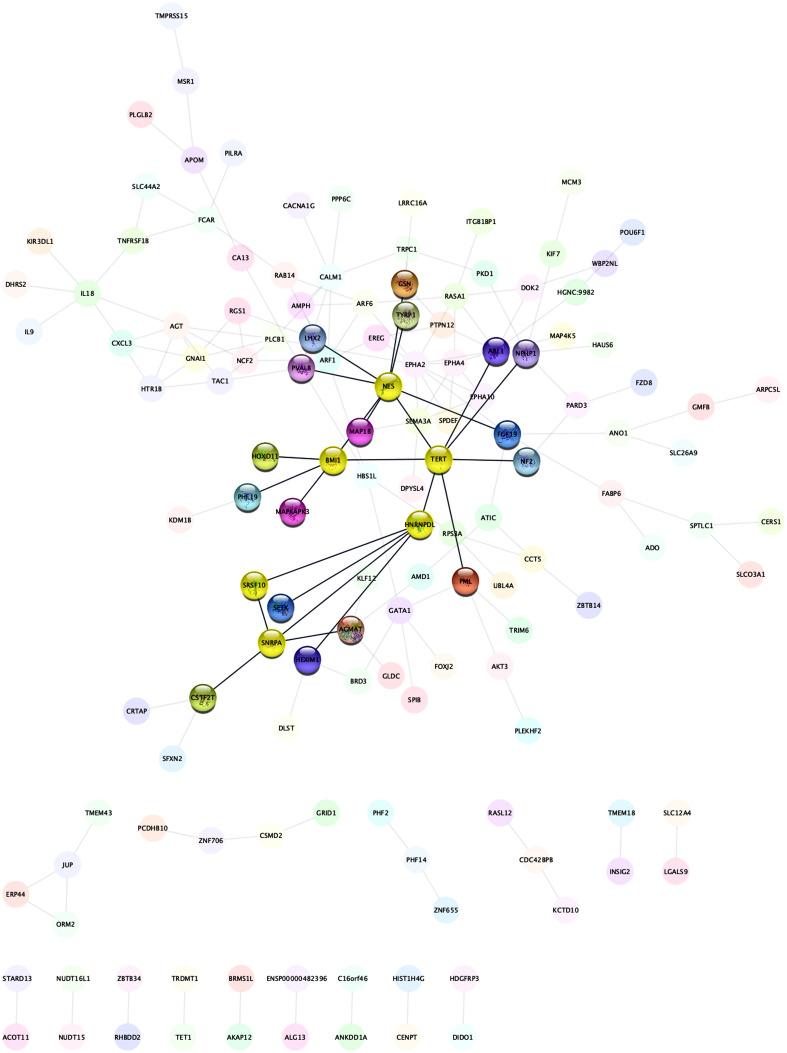
Protein–protein interaction network of differentially expressed genes.

**Figure 5.**
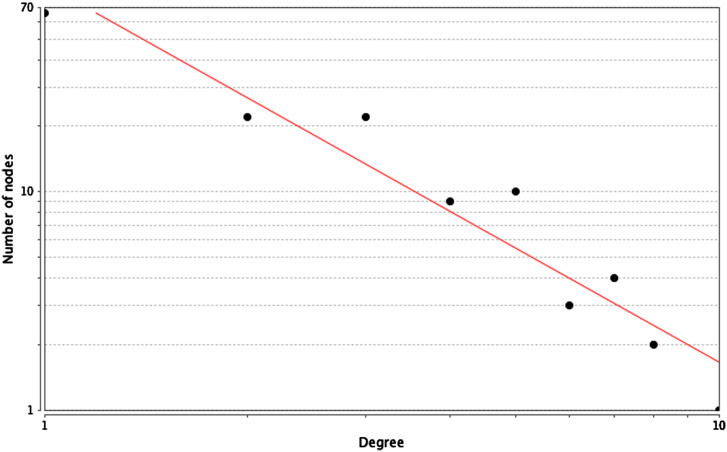
The distribution of core genes in the interaction network. The black node means the core gene. The red line means the fitted line and the blue line means the power law. The correlation between the data points and corresponding points on the line is approximately 0.981. The *R*-squared value is 0.916 giving a relatively high confidence that the underlying model is indeed linear.

**Figure 6.**
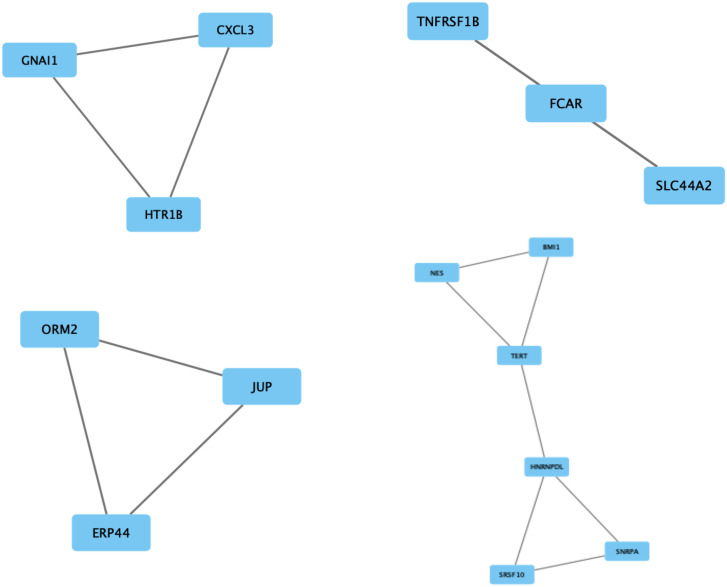
The top 4 modules from the gene–gene interaction network. The squares represent the differentially expressed genes (DEGs) in modules, and the lines show the interaction between the DEGs.

STRING data revealed a gene–gene interaction network comprising 231 nodes (DEGs) and 167 edges (interactions), and a PPI network was constructed (Figure 4). The 10 main high-degree hub nodes encompassed *EPHA2*, *ABL1*, *NES,* *TERT*, *AGT*, *CALM1*, *ARF1*, *RASA1*, *EPHA4*, and *IL18*. Of the abovementioned genes, *EPHA2* had the highest node degree of 10. Table 3 shows core genes and their respective degrees. Figure 5 depicts the overall prospect of the complex regulatory relationship among the detected core genes. The data points and the respective points on the graph were highly correlated (coefficient approximating 0.981). An *R* ^2^ of 0.986 was obtained, indicating the linearity of the model. Next, MCODE was utilized for screening modules in the gene interaction network, 4 of which are depicted in Figure 6. The first module comprising *CXCL3*,* HTR1B*, and* GNAI1* had a score of 3, with 3 nodes and 3 edges. The second module also had 3 nodes and 3 edges and encompassed *FCAR*, *SLC44A2*, and *TNFRSF1B*, with a score of 2. The third module (*JUP*, *ORM2*, and *ERP44*) has a score of 2, and included 3 nodes and 3 edges. Finally, the fourth module (*SRSF10*, *SNRPA*, *HNRNPDL*, *TERT*, *BMI1,* and *NES*) had a score of 2, with 6 nodes and 7 edges.

### Validation of Prognostic Factors

The univariate Cox regression analysis revealed that in the entire cohort, the age [hazard ratio (HR) = 1.442, 95% CI: 0.705-2.951, *P* = .314], gender (HR = 1.687, 95% CI: 0.820-3.469, *P* = .151), size (HR = 1.171, 95% CI: 0.568-2.413, *P* = .668), tumor stage including T (HR = 1.565, 95% CI: 0.819-2.988, *P *= .176), N (HR = 1.470, 95% CI: 0.707-3.057, *P* = .299), M (HR = 1.357, 95% CI: 0.321-5.732, *P *= .677), localization of tumor (HR = 1.485, 95% CI: 0.692-3.185, *P* = 0.307), vessel invasion (HR = 1.067, 95% CI: 0.254-4.490, *P *= .929), differentiation (HR = 2.167, 95% CI: 1.052-4.462, *P *< .05), and hub genes such as *JUP* (HR = 1.385, 95% CI: 0.909-2.111, *P* = .137), *CALM1* (HR = 1.401, 95% CI: 0.901-2.178, *P* = .132), *NES* (HR = 1.000, 95% CI: 0.657-1.523, *P* =.999), *EPHA2* (HR = 1.225, 95% CI: 0.890-1.687, *P* = .213), *ARF1* (HR = 1.196, 95% CI: 0.785-1.822, *P* = .404), *ORM2* (HR = 1.187, 95% CI: 0.796-1.772, *P* = .400), *TERT* (HR = 1.203, 95% CI: 0.827-1.749, *P* = .333), *IL18* (HR = 1.107, 95% CI: 0.781-1.570, *P* = .568), *CXCL3* (HR = 1.135, 95% CI: 0.783-1.644, *P* = .504) were related to PNI prognosis in PDAC, and differentiation (HR = 10.919, 95% CI: 2.039-58.482, *P* = .005) remained the independent predictor via multivariate Cox regression analysis (Table 4). These outcomes pointed out that differentiation could be an independent predictor of PNI prognosis in PDAC.

### Validation of Quantitative Real-Time Polymerase Chain Reaction

**Figure 7.**
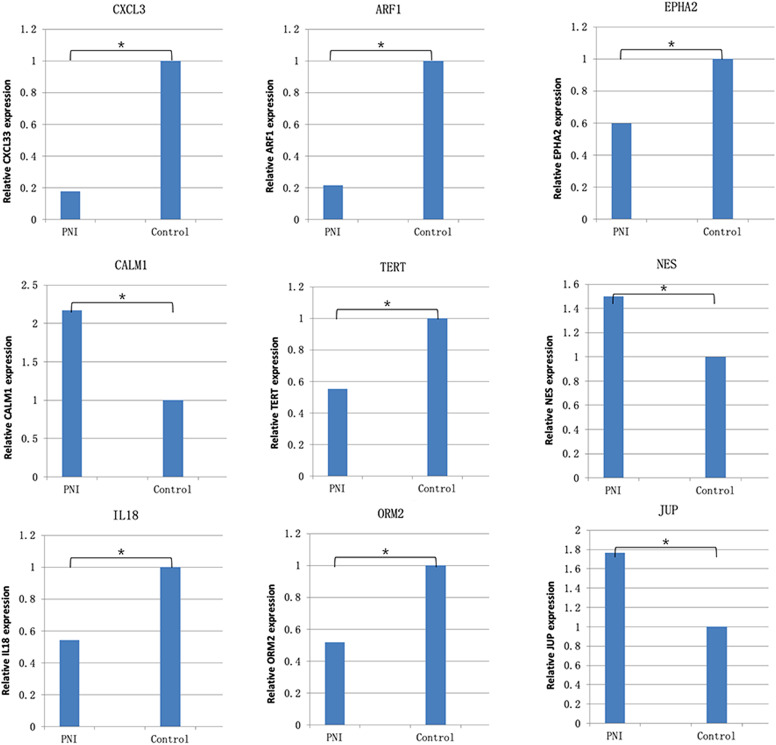
Results of the quantitative real-time PCR. Perineural invasion (PNI) groups: mRNA levels of pancreatic ductal adenocarcinoma (PDAC) tissue samples from patients diagnosed with PNI; control groups: mRNA levels of PDAC tissue samples from patients diagnosed without PNI. Asterisk means *P < .*05.

In addition to validating the bioinformatic analysis results, quantitative RT-PCR was used to quantify parts of explored genes, including 3 upregulated genes (*JUP*, *CALM1*, and *NES*) and 6 downregulated genes (*EPHA2*, *ARF1*, *ORM2*, *TERT*, *IL18*, and *CXCL3*). As shown in Figure 7, the gene expression patterns of *JUP*, *CALM1*, *NES*, *EPHA2*, *ARF1*, *ORM2*, *TERT*, *IL18*, and *CXCL3* detected by quantitative RT-PCR significantly were accorded with the corresponding gene alteration of microarray data (*P < .*05).

### Survival Analysis

**Figure 8.**
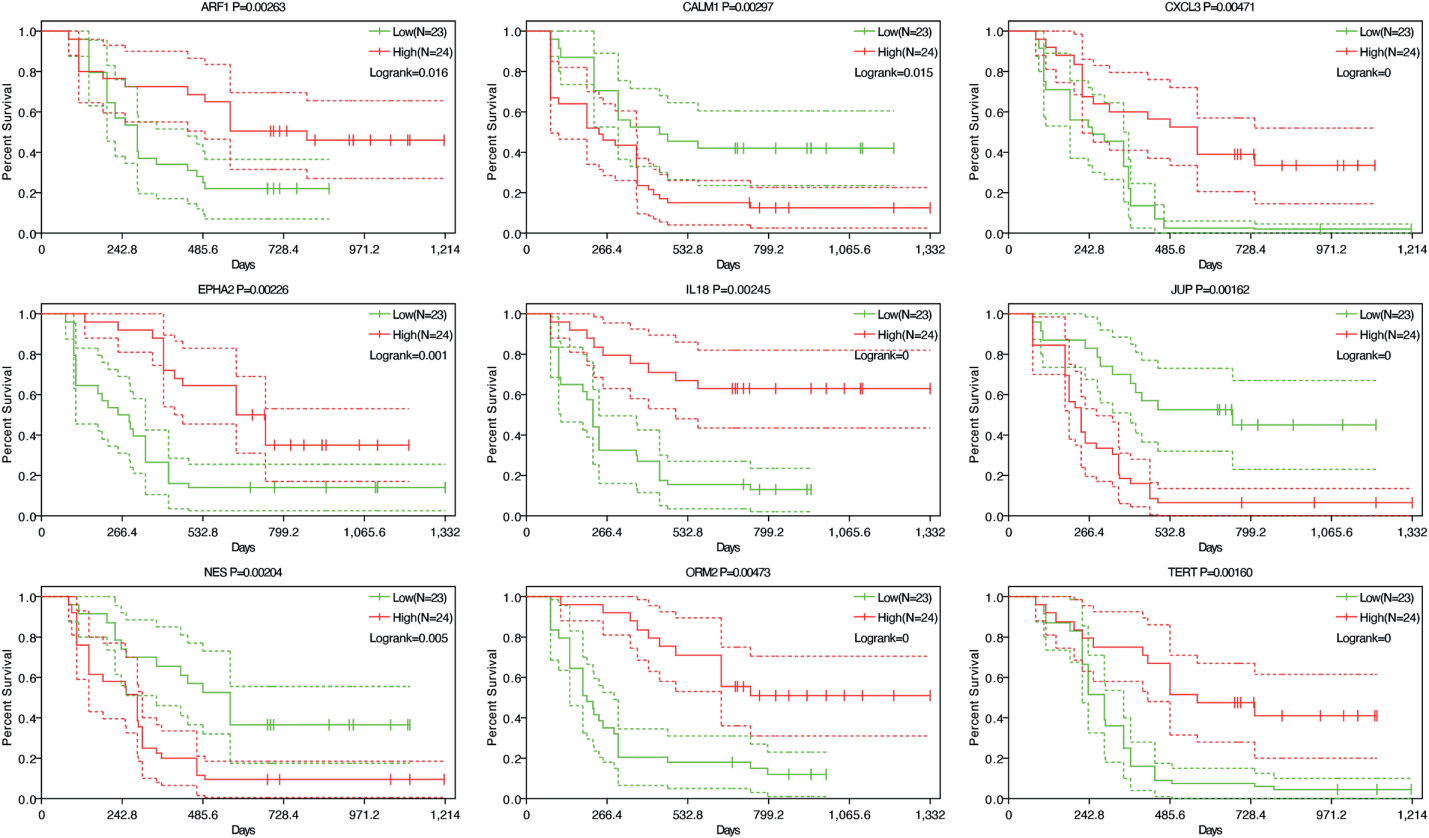
Overall survival analyses 9 hub genes in data set GSE102238.

Cases in the GSE102238 dataset were assigned to 2 groups, based on the median expression levels of various hub genes and Kaplan–Meier survival analysis. For the hub genes *JUP*, *CALM1*, and *NES*, respectively, individuals with elevated gene expression had markedly worse OS (*P* < .05). For the hub genes *EPHA2*, *ARF1*, *ORM2*, *TERT*, *IL18*, and *CXCL3*, respectively, individuals with lower gene expression showed significantly worse OS (*P* < .05; Figure 8). These findings suggested key roles for these genes in PNI prognosis in PDAC.

## Discussion

Neural invasion represents a critical invasion pathway in PC. Recent evidence indicates neural invasion-related genes in PC play sequential roles, via multiple pathways, involving specific growth factors, adhesion molecules, matrix metalloproteinases, and other effectors; then, these genes undergo changes in the generation, and tumor cells subsequently invade the nerve tissue.^17,18^ Zhang et al’s^19^ study identified PNI-associated genes in PC cell lines and the “chemokine signaling pathway” was found to be associated with PNI, following KEGG pathway enrichment analysis and the construction of a PPI network from the identified DEGs. Furthermore, FGF2 was found to be associated with PNI. Li et al’s^20^ review showed gene alternations in human PDAC samples are also linked to PNI. For example, Ras homolog family member C was abundantly expressed in PNI tissues and was related to poor disease prognosis. Here, GSE102238’s gene profiling data were obtained and analyzed by bioinformatics. As shown earlier, 242 DEGs were identified in PDAC tissue specimens from patients diagnosed with PNI compared with PDAC tissue samples from patients without PNI. In addition, enrichment analysis and gene interaction network analyses were carried out for identifying biomarkers or key genes associated with cytogenetic pathways controlling PNI in PDAC. Univariate Cox and multivariate Cox regression analyses were used to identify the most significant prognostic factors and genes. Finally, the differentiation was identified as an independent prognostic factor (*P < .*05) and 3 upregulated genes (*JUP*, *CALM1*, and *NES*) and 6 downregulated genes (*EPHA2*, *ARF1*, *ORM2*, *TERT*, *IL18*, and *CXCL3*) were not statistically significant by univariate Cox analysis but were validated by quantitative RT-PCR. Overall survival analysis suggested key roles for these 9 hub genes in PNI prognosis in PDAC.

To explore the underpinning mechanisms linking PNI and PDAC, retained GO functions and signaling pathways involving the detected DEGs were examined. Based on GO analysis, DEGs were shown to be involved in methylated histone binding, chemokine, transmembrane receptor protein tyrosine kinase, transmembrane-ephrin receptor, phosphoric diester hydrolase, and phospholipase C activities, as well as positive regulation of oxidoreductase and monooxygenase activities, which likely play critical roles in PNI development in PDAC. Corroborating previous findings, KEGG pathway analysis in this study demonstrated the NF-κB, VEGF, PI3K/AKT, and other pathways were critical for PNI development in PDAC. NF-κB is important in the pathogenesis of PC, inducing epithelial–mesenchymal transition and invasion-related factors. Further, we demonstrated the critical effects of NF-κB +mediated neural-tumor co-culture invasion as well as dorsal root ganglia neural outgrowth by disrupting the tumor-neural cross-talk. In experimental animals, Minnelide decreases neurotrophin biosynthesis, nerve density, and sciatic nerve invasion. The critical role of the NF-κB pathway in PC progression, via EMT promotion and lymphovascular and neural invasion, was also demonstrated.^21^ In the in vitro experiment of mouse dorsal root ganglion in prostatic cancer, nerves injured by tumor invasion promote the release of CCL2, generate an inflammatory reaction of nerve restoration, and induce CCR2 cancer cells to migrate to these nerves. By activating the MAPK and Akt pathways in pc3 cells, they promote the occurrence of PNI.^22^ Overexpressed CXCR4 in bile duct carcinoma and PC is closely related to PNI, lymphatic metastasis, TNM staging, and vessel invasion. CXCR4 promotes VEGF expression, the mitosis and proliferation of vascular endothelial cells, and the tumor to generate new vessels. It also promotes the specific growth tendency of axons and increases nerve–tumor contact.^23-26^ The abovementioned pathways may have critical functions in PNI development in PDAC.

It should be noted that multiple reports suggested the abovementioned DEGs in PDAC tissue specimens from patients diagnosed with PNI are critical for PNI occurrence in PDAC. The STRING database identified the top 10 high-degree hub nodes of DEGs, i.e., *EPHA2*, *ABL1*, *NES*, *TERT*, *AGT*, *CALM1*, *ARF1*, *RASA1*, *EPHA4*, and *IL18*. Next, the gene interaction network and top 4 modules were examined with MCODE, and *CXCL3*, *HTR1B*, *GNAI1*, *FCAR*, *SLC44A2*, *TNFRSF1B*, *JUP*, *ORM2*, *ERP44*, *SRSF10*, *SNRPA*, *HNRNPDL*, *TERT*, *BMI1*, and *NES* were detected as core interaction genes, which might constitute targets for treating PNI in PDAC. Of these, *TERT* and *NES* corroborated STRING database findings.

Nestin (NES) is a class VI intermediate filament protein, which is distributed in the cytoplasm and involved in the formation of the cytoskeleton. It was previously considered as a marker of neural stem cells. In recent years, it has been found that nestin is expressed in pancreatic stem cells and PC stem cells and is related to tumorigenesis, tumor angiogenesis, tumor metastasis, and prognosis of PC.^27^ The neural invasion pathway of PC is usually through the direct destruction of the perineural membrane, the vascular invasion of the perineural membrane, and the destruction of the synaptic membrane of the nerve endings.^28^ In this study, we focused on analyzing the perineural invasiveness of PDAC based on nestin expression in cancer cells. Kawamoto and collaborators as well as other investigators revealed nestin amounts in tumor cells correlate with nerve invasion in PC.^29^ Furthermore, nestin is strongly immunoreactive in nerve fibers of both PDAC and chronic pancreatitis specimens, likely indicating neural remodeling that is critical for the generation of pancreatic neuropathy.^30^ The *TERT* gene at chromosome 5p15.33 is translated into the catalytic subunit of telomerase reverse transcriptase that represents a constituent of the protein/RNA complex maintaining telomere ends. Bao et al^31^ found that pre-diagnostic leukocyte telomere length and genetic alterations in TERT alter PC risk. Faleiro et al^32^ found that TERT hypermethylated oncologic region is hypermethylated in pancreatic tumor tissue when compared with normal tissue and that TERT hypermethylated oncologic region methylation correlates with TERT expression in tumor samples, which supports the diagnostic and prognostic values of TERT hypermethylated oncologic region in PC. In agreement, Campa et al^33^ reported the TERT locus alters PC risk, likely via multiple independent variants. Jointly, the above core genes detected in PDAC tissue specimens from patients diagnosed with PNI by bioinformatics and gene interaction network analyses might control PNI capacity in PDAC.

Some of the genes and pathways described in the present report should be further researched to develop therapeutic targets for PNI in PDAC. Tumor angiogenesis constitutes a critical parameter affecting proliferation, invasion, metastasis, and drug sensitivity in PDAC. This could be explained by the fact that elevated amounts of tumor vessels increase the odds of cancer cells to enter the bloodstream. New tumor vessels or capillaries possess weak and leaky basement membranes, and cancer cells could penetrate them more readily compared with mature counterparts. Furthermore, cancer cells can directly invade the nerve and can also invade the nerve through the penetrating channels (such as blood vessels and reticular fibers).^33^ Our result also discovered that the VEGF signaling pathway was one of the most relevant pathways for PNI in PDAC. So nestin may mediate increased PNI in PDAC by raising tumor cells invading the nerve through the blood vessels and activating the VEGF signaling pathway. Nestin could act as a novel therapeutic target for PC via tumor angiogenesis.

Our study identified 242 DEGs including 68 upregulated DEGs and 174 downregulated DEGs screened in PDAC tissue from patients diagnosed with PNI compared with PDAC tissue from patients without PNI. Bioinformatics analysis and quantitative RT-PCR showed that parts of DEGs including *JUP*, *CALM1*, *NES*, *EPHA2*, *ARF1*, *ORM2*, *TERT*, *IL18*, and *CXCL3* were mainly linked to the understanding of the molecular mechanisms between PDAC and PNI. The NES/VEGF signaling pathway may be a promising approach to analyze the development process of PNI on the microenvironment of PC. Univariate and multivariate Cox analyses and OS analysis suggested the differentiation as an independent prognostic factor and key roles for these 9 hub genes in PNI prognosis in PDAC.

## Figures and Tables

**Table 1.** The Top 10 Regulated Differentially Expressed Genes in Pancreatic Ductal Adenocarcinoma Tissues with Perineural Invasion and Without Perineural Invasion with *P* < .05

ID	*P*	Log FC	Gene Symbol
Upregulated			
A_24_P472455	.0097106	1.0600455	*ARF6*
A_24_P115511	.0047033	0.9860812	*RAB14*
A_23_P103672	.0059544	0.8413052	*NES*
A_23_P19369	.0445384	0.8021201	*CARMIL1*
A_23_P108501	.0290841	0.7675065	*EPHA4*
A_24_P29594	.0121245	0.7621006	*HBS1L*
A_24_P235305	.0325074	0.756461	*ZNF706*
A_23_P375147	.0471249	0.734487	*RC3H2*
P14923	.0436128	0.702987	*JUP*
A_23_P32036	.0224597	0.6723084	*NMRK1*
Downregulated			
p26684	.002203	−1.6538247	*IGHV1-2 *
P19652	.014338	−1.2538799	*ORM2*
A_23_P324754	.004661	−1.2253182	*MESD*
A_24_P100830	.033902	−1.1305032	*AMN1*
A_24_P385585	.0297	−1.0359545	*TMEM18*
P29317	.0297	−1.0246753	*EPHA2*
A_23_P314115	.026752	−1.023961	*BMI1*
A_33_P3401008	.025179	−0.9533247	*TMEM150B*
A_23_P335495	.025179	−0.9526981	*ANO7*
A_23_P333852	.03207	−0.9288766	*TTLL11*

DEGs, differentially expressed genes; FC, fold change; PDAC, pancreatic ductal adenocarcinoma; PNI, perineural invasion.

**Table 2.** Core Pathways and Their Associated Genes Found

GO ID	GO Term	Term *P*	% Associated Genes	Associated Genes Found
R-HSA:5218920	VEGFR2-mediated vascular permeability	.01	10.34	[*AKT3*, *CALM1*, *JUP*]
R-HSA:8863795	Downregulation of ERBB2 signaling	.01	10.34	[*AKT3*, *EREG*, *PTPN12*]
R-HSA:3928663	EPHA-mediated growth cone collapse	.01	10.00	[*EPHA10*, *EPHA2*, *EPHA4*]
R-HSA:5339562	Uptake and actions of bacterial toxins	.01	8.11	[*ANTXR2*, *CALM1*, *SV2B*]
R-HSA:4420097	VEGFA-VEGFR2 Pathway	.00	6.06	[*AKT3*, *CALM1*, *JUP*, *MAPKAPK3*, *NCF2*, *RASA1*]
R-HSA:1227986	Signaling by ERBB2	.03	6.00	[*AKT3*, *EREG*, *PTPN12*]
R-HSA:3928665	EPH-ephrin-mediated repulsion of cells	.03	5.88	[*EPHA10*, *EPHA2*, *EPHA4*]
R-HSA:194138	Signaling by VEGF	.00	5.61	[*AKT3*, *CALM1*, *JUP*, *MAPKAPK3*, *NCF2*, *RASA1*]
R-HSA:112043	PLC beta-mediated events	.03	5.56	[*CALM1*, *GNAI1*, *PLCB1*]
R-HSA:112040	G-protein-mediated events	.03	5.45	[*CALM1*, *GNAI1*, *PLCB1*]
R-HSA:2682334	EPH-Ephrin signaling	.03	4.35	[*EPHA10*, *EPHA2*, *EPHA4*, *RASA1*]
R-HSA:446193	Biosynthesis of the N-glycan precursor (dolichol lipid-linked oligosaccharide, LLO) and transfer to a nascent protein	.08	3.85	[*ALG13*, *ALG9*, *ST6GALNAC6*]
R-HSA:425393	NF-κB signaling pathway	.05	3.70	[*CALM1*, *SLC12A4*, *SLC26A9*, *SLC9A8*]
R-HSA:2672351	Stimuli-sensing channels	.05	3.67	[*ANO1*, *ANO7*, *CALM1*, *TRPC1*]
R-HSA:8876198	RAB GEFs exchange GTP for GDP on RABs	.11	3.33	[*AKT3*, *RAB14*, *TRAPPC13*]
R-HSA:111885	Opioid signaling	.11	3.30	[*CALM1*, *GNAI1*, *PLCB1*]
R-HSA:9007101	Rab regulation of trafficking	.08	3.23	[*AKT3*, *ARF6*, *RAB14*, *TRAPPC13*]
R-HSA:2219528	PI3K/AKT signaling in cancer	.14	3.00	[*AKT3*, *EREG*, *FGF19*]

GO, Gene Ontology.

**Table 3.** The Core Genes and Their Corresponding Degree

Gene	Degree	Gene	Degree	Gene	Degree	Gene	Degree
*GAPDH*	56	*RPS3A*	32	*EIF3b*	28	*RPL18*	25
*GART*	41	*EIF4E*	31	*DDX5*	28	*CALM3*	25
*FAU*	39	*MAPK3*	31	*HSPD1*	28	*ACTG1*	25
*HSPA8*	38	*IL6*	29	*RPS29*	26	*RPS27*	24
*EEF1A1*	36	*RPL6*	28	*RPL18A*	26	*RPL32*	24

**Table 4.** Cox Regression of the Clinical Characteristic and the Hub Genes

Variables	Univariate Cox Analysis		Multivariate Cox Analysis	
	HR (95% CI)	*P* by Log Rank	HR (95% CI)	*P*
Age	1.442 (0.705, 2.951)	.314	–	
Gender	1.687 (0.820, 3.469)	.151	–	
Sizes	1.171 (0.568, 2.413)	.668	–	
T stages	1.565 (0.819, 2.988)	.176	–	
N stages	1.470 (0.707, 3.057)	.299	–	
M stages	1.357 (0.321, 5.732)	.677	–	
Localization of tumor	1.485 (0.692, 3.185)	.307	–	
Vessel invasion	1.067 (0.254, 4.490)	.929	–	
Differentiation	2.167 (1.052, 4.462)	.032	10.919 (2.039, 58.482)	.005
*JUP*	1.385 (0.909, 2.111)	.137	–	
*CALM1*	1.401 (0.901, 2.178)	.132	–	
*NES*	1.000 (0.657, 1.523)	.999		
*EPHA2*	1.225 (0.890, 1.687)	.213		
*ARF1*	1.196 (0.785, 1.822)	.404		
*ORM2*	1.187 (0.796, 1.772)	.400		
*TERT*	1.203 (0.827, 1.749)	.333		
*IL18*	1.107 (0.781, 1.570)	.568		
*CXCL3*	1.135 (0.783, 1.644)	.504	-	

HR, hazard ratio.

**Supplementary Table 1.** Primers of Genes Validated by qRT-PCR

Gene	Forward	Reverse
JUP	5′-TCGCCATCTTCAAGTCGGG-3′	5′-AGGGGCACCATCTTTTGCAG-3′
CALM	5′-GGTTGGAGATGTTGAGGCTGAT-3′	5′-ATGGTGCCATCGCCATCTTT-3′
NES	5′-GAAGGGCAATCACAACAGGTG-3′	5′-GGGGCCACATCATCTTCCA-3′
EPHA2	5′-ACTACGGCACCAACTTCCAG -3′	5′-GTAGAAGCCTTTGCGGGTGA-3′
ARF1	5′-GGAGCAAAACCAACGCCTG-3′	5′-GGCCAGGGACACCTCAAG-3′
ORM2	5′-AGTACCAGACCCGCCAGAAC-3′	5′-CTAAGGAACAGCAGGTGAGCA-3′
TERT	5′-AAACCTTCCTCAGCTATGCCC-3′	5′-GTTTGCGACGCATGTTCCTC-3′
IL18	5′-CAACTCTGGCTGCTAAAGCG-3′	5′-AGCCATCTTTATTCCTGCGAC-3′
CXCL3	5′-CCAAACCGAAGTCATAGCCAC-3′	5′-TGCTCCCCTTGTTCAGTATCT-3′

**Supplementary Table 2.** The Complete Regulated DEGs in PDAC Tissues with PNI and without PNI with *P* < .05

ID	adj.P.Val	*P*	t	B	logFC	SEQUENCE
ARF6	0.999	.0097106	2.684196	-3.84	1.0600455	AGAGGAGATAAATCTCCTTGTTATCACAATAGTTAATGTGTCATGATCACTGACTAACTG
RAB14	0.999	.0047033	2.95293	-3.67	0.9860812	GGACGTGCTTCTACAAGAACAGTCCTGAGTCCACGTTCTGTTTAGCTTTAGGAAGAAACA
NES	0.999	.0059544	2.867092	-3.72	0.8413052	CACGGCCACCGCCTCTTTTGTTAACAGGACAGTTGATCCATTAAATTAAAAATCATTCAA
CARMIL1	0.999	.0445384	2.058427	-4.19	0.8021201	TAGCCAAACAACAGTTTTGGATTCACTGACTGATTATGAAAGAAGCAGTAGACTGGTATC
EPHA4	0.999	.0290841	2.243988	-4.09	0.7675065	TAGTCCACAACGCTTATCGATTTGTGTTGCTGCAACATGCTAGTGTACATTTTATAATAA
HBS1L	0.999	.0121245	2.598743	-3.89	0.7621006	GATCCTGCTACCAGGTTATGATAGATTTTATGGTATGTCTCAAGATATTGAGATAAAGGT
ZNF706	0.999	.0325074	2.196459	-4.12	0.756461	TAGTCCACAACGCTTATCGATTTGTGTTGCTGCAACATGCTAGTGTACATTTTATAATAA
RC3H2	0.999	.0471249	2.033087	-4.21	0.734487	TACCTCACCACAAAGGAGCTGCTGAGCTCCTGGCTGCAAAGTGACGATGAACCGGAGAAG
JUP	0.999	.0436128	2.067805	-4.19	0.702987	TCTCTGGATGGAGAATTCCACAGCTGATTGGAACCTAAACGAGAGAACCAAATGGACATC
NMRK1	0.999	.0224597	2.352108	-4.03	0.6723084	ACTGTGGTACAATGACAGCCATTGTTTCATATGTTTGATTTTTATTGCACATGGTTTTCC
ZCCHC24	0.999	.0381761	2.126677	-4.16	0.6678506	TGTTCCAAAAAGAATTCCAACCGACCAGCTTGTTTGTGAAACAAAAAAGTGTTCCCTTTT
SLC26A9	0.999	.0072626	2.793656	-3.77	0.6591136	GGTTCAACTTGCTCATCTGATGTGTGAATTACATCACACAGTTGAATGAGAATTTGTTAA
DOK6	0.999	.0248178	2.310709	-4.06	0.6583766	TGAGACCAATCCAGTATTTCAGCCTTGTTTGCTTTGCAATTACGGGTTGGCTTGTGGCTA
EPHA10	0.999	.0231251	2.340047	-4.04	0.6571396	AATAAAGCATGTGAGGTTAGCACACCGTATATTCTCAAATCCTGGAGCTACTGGTCCTCT
AMPH	0.999	.0244336	2.317207	-4.05	0.6546721	ACTGTGGTACAATGACAGCCATTGTTTCATATGTTTGATTTTTATTGCACATGGTTTTCC
ANKDD1A	0.999	.0149145	2.517508	-3.94	0.6465227	AGATGCACGGCAGAATGGTTCCCGTCTGAGCCAGTACTGAATAAACTCAAAACTCTTGAA
CNTNAP3B	0.999	.03366	2.181448	-4.13	0.6364675	GCGCCATGGCAAAAGCAAATTTAGACATTTTTAAAAGGAAACAGATTCTAGGATGTACAA
ZNF534	0.542	.0000213	4.671202	-2.46	0.6296364	AAGCAGCTTGTATAATTCCAACTGGTGTTTCATTTCTGTTCTAATGCTAAGTGGTAACGC
LHX2	0.999	.0196994	2.405829	-4	0.6247175	TCAAATGTTCCCCCTCAGGTTATTTTGCTTATGGTACCCATGAGTTGCCTCTCTCTGTAC
WBP2NL	0.999	.0189837	2.420858	-3.99	0.6216851	GCACAGTGTGGAAGAACTCAACTTTTTAAAGACAGAGATCAGCTTTAAATATTGCTAAGA
ITIH5	0.999	.01888	2.423079	-3.99	0.61875	AGCTACAAAGCATGGGAAAAAGAGACTCTTTTAGGATCAGATCTGTGAGCACGTTGGCGA
TYRP1	0.999	.0460494	2.043473	-4.2	0.6114545	ATGAGTGATCTAAATTTGCAGCAATGATACTAAACAACTCTCTGAAATTTCTCAAGCACC
globoside blood group)(B3GALNT1	0.999	.0105033	2.654176	-3.86	0.6098182	GCATTTGGCTGCATAGCTACAATGATGGCATTTTAACTTATTCATGATTAGAGATGATGA
ZFP37	0.999	.0128818	2.575138	-3.9	0.5967565	CCCAGCAGCATGCTTTGTACACTGATATATTGGGTAAATTTTGTTGAATAAATTAAGCTC
GATA1	0.999	.0124498	2.588443	-3.9	0.5952825	CCTGTTTCCATTTGAAAGGAACTGTAAGCTTTTATCTTTTAACCAACTGAACAATACACC
TRIM56	0.999	.0266215	2.281346	-4.07	0.5885617	GCCAGAGCTTAATCCTTGATGTCCTACTGATAAGGTTTGCATTCTAACAACACATGTAAA
CALM1	0.999	.0121591	2.597637	-3.89	0.5864026	AGGAGCAGGCTGGGATCCCAACTATCGCTTGTTGCCTCTTTTTCAAGTGGAATTTGAATT
ARHGAP19	0.999	.0307192	2.220703	-4.11	0.5843442	TGTTTCACAGTACAGGATCTGTACATAAAAGTTTCTTTCCTAAACCATTCACCAAGAGCC
BZW1P2	0.999	.0238343	2.327529	-4.05	0.5811429	GTGTTCAAAGCCCACTTTAGAACCTAAAGCATTTTGGGTTAATAAAGCAAAATTTTGCTT
TNFRSF1B	0.999	.0360703	2.151465	-4.14	0.5809545	GACGTTGGTATAACATTGCTTCTTGAAAAATGAAAGTATTTGGACATACAGACAGAAGCA
CCT5	0.999	.0291124	2.243575	-4.09	0.5779123	AAGCCTGGCTATCCTGGAGTTTTCCAGTTCATCAGCCATCCAATAAAAATGATGTCAAGC
ABL1	0.999	.0329891	2.19013	-4.12	0.5775682	CAGCTTTAAATAGACTTTGTCATATGCATGAATCATCAGAGATGAAACTGTTTGAGAGAC
POU6F1	0.999	.0133427	2.561381	-3.91	0.5698669	TCATTCTGCGACACTGGCCATAGCTGAGCAACATGAAAGATTAATGCTAGAAATGGAACA
LY6E-DT	0.999	.0148401	2.519485	-3.94	0.5691656	GGATCCTGCTACCAGGTTATGATAGATTTTATGGTATGTCTCAAGATATTGAGATAAAGG
KDM1B	0.999	.0104999	2.654303	-3.86	0.5691494	CTGTCTCCTGTTTTCAACACATTCCATGGAATAAATTCCTTATTGAAGGTAACTTTCAGA
ZNF510	0.999	.033621	2.181949	-4.13	0.5672922	AGAGGCGTGTTACACCGTATCTGTTCTATCCTCATCTAAATACTGAGAGGCTAGTTGGGA
FOXJ2	0.999	.0334561	2.18407	-4.13	0.566289	CAAATATTGAAGAATCTCTAACCAGGGACACCAGTCCCTACGAAGACCTTGGGCGATTTT
G2E3	0.999	.0027109	3.148241	-3.54	0.5656039	TGAGTAATGTACAGCCTCCTACTTTGATTAACCTAGCATTTATTCCCTCTTCTGGATCAG
CSTF2T	0.999	.0175426	2.452738	-3.98	0.5565617	ACTACCTTCACTGGCATTTCCATAGTCCTGGAATCCAGAGCCAAGTGGCCTATCTAAAAT
TULP2	0.999	.0066449	2.826657	-3.75	0.5546169	ACAACTTGCAGCTCATCTCTATTGATGGTGAAACAGTCGCAAATGGAATTCCAAGAAACA
TET1	0.999	.0298185	2.233391	-4.1	0.5539968	GAGGACAGGCACTTACACTAATCTGGAAGCATAATATATAAAGAGTACCTACAAATCAAT
PBDC1	0.999	.0321921	2.200647	-4.12	0.5501039	ACCTGGTTCTTAAGCAAATTCTAGATTGGAACAATTAGAAGATCTGACTGATGTTAAGAG
class V)(ADH6	0.999	.0354967	2.158438	-4.14	0.547	AGGTTAGTCTTTCAGTACACGTTACTGGTAAGTAGTTTCCAAGTTACGTGTTGTCACTGG
ZNF92	0.999	.0309732	2.217184	-4.11	0.5467987	AAATTTATTACTCAGATTCTACCCTGTCAACCCAGGACGTGTTACTTACACATTTCCCAG
PYCR2	0.999	.0452436	2.051394	-4.2	0.5440227	CGTGTTCTGTGTTAGTGATCACTGCCTTTAATACAGTCTGTTGGAATAATATTATAAGCA
PVALB	0.999	.0330448	2.189402	-4.12	0.5403312	AATGAATTCTTCTTAACCTATCCATCTTTTGTGCCAGATACATGCAGTACCTACACCCCA
FGF19	0.999	.0081819	2.74905	-3.8	0.5393084	CACCAGCAGTAATATGGGAAGCTATGCTGAAAACCGCTATTTTGAATAATGTGAAATAAA
KCNJ13	0.999	.0394035	2.112775	-4.16	0.5386688	GCAAGATCCCCCGATCAGCTTTATCTGCCATTGTCAATGGCAAGCCATACAAGATAACTT
CACNA1G	0.999	.036255	2.149242	-4.14	0.5379123	CTGCATTTTTCCCCATAAAAACCAGTGTTTTGTATTTGTAAGAATGTTGCTGTGTAATCC
ADO	0.999	.0201129	2.397367	-4.01	0.5352532	ACAGGACTTTTAGTTGTATCACCTCAAGAGATTTTGAAGTTTGTGATCAAGGTCTGTATA
TLL2	0.999	.0405136	2.100524	-4.17	0.5317695	GTTCTAGTTTCCCGGCATTGATAGTTCCCTATTTGAAATATAATGTTTCTCTTGTAAGTG
LMCD1	0.999	.0201444	2.396731	-4.01	0.528526	AAACTTTGGAGAGGGAAAATCTTCACTTTCTTAAGCAACAATGGATATTGCCTGTGTTTG
CAMSAP1	0.999	.0009466	3.504242	-3.3	0.5268864	TTGCCTCTAGAGAACACATTCCTCCTATTCTGGGGTCCCGTGAGAGAAAGAAATGCTTTT
KIR3DL1	0.999	.02819	2.257216	-4.09	0.5267273	CCGGATGCACTGGGGCTATCTAACAGTACTGGCATCTGATAGGTAGAGGTCAGGTACGCT
TOR1B	0.999	.0160322	2.488791	-3.95	0.5232825	GAATATTAAGTGCTACTTGAGGTACATGTTCAGACTAACATTCTTTTGCAGTATAGTGAG
C17orf67	0.999	.0241401	2.322233	-4.05	0.5213766	GCCCTGACTATTGTAAGAGGTAAACTTACCTGGTTTGTTTGAGAATGACCATTTTCCTAA
ITPRIPL2	0.999	.0229187	2.343755	-4.04	0.5202273	AGGGACAGAGAAGATCAGCTGCAGACAACTGAGGAATCCTTTGCAAAGGTTAGTGTATAA
ETFRF1	0.999	.0306162	2.222137	-4.11	0.5192013	GTACCAGTTTAAGAAGTGAGAACTTCTTCACTGACTGAAATTTGCATATCAATCCAAATC
CRTAP	0.999	.050964	1.997608	-4.22	0.5159448	GCATTTCAAGCTCATTCTAAGTTCATCTTATCAATGACAAGAGGAGACACGTTGTTCATT
TSPYL1	0.999	.0287197	2.249335	-4.09	0.5158766	CTCAGCAATCTTTCGTTCTAGTTATATTCGGTCTTTGAAACTGACAATCTTTGAAATGTG
TRDMT1	0.999	.0139146	2.544898	-3.92	0.5141981	TGTGTAGTTATGGATACAGAAGAAGATAACAAACATGTAGGTCATCTTCTTGAAGAAGTG
CCDC40	0.999	.032026	2.202868	-4.12	0.5134513	TAGGCTTCTTAGTAGCAGCTTTGTACACTGAGGACACTGTAGCCAGGAACCTGTGCATGC
PLCB1	0.999	.018563	2.429935	-3.99	0.5129773	GCAGAGAAATGTCAAGCTTTCCCTTAAAAACAAAAAACAAAAGCAAACCCTCACATCTCC
SPTLC1	0.999	.0270766	2.274217	-4.08	0.5074935	TCAAAATCAGTGATGGGAGTAAGAGCAAATTTCATCTTTCCAAATTGATGGGTGGGCTAG
GSN	0.999	.0496015	2.009927	-4.22	0.5024123	ATTACTGTCTGTGAGAGTTACTACTTTGTAACTTATGGTTTCTGCTTCAGTATTGTGTTG
MAP1B	0.999	.0228701	2.344632	-4.04	0.5019481	CACATGTAACAGATTCCTTTATATGTAGTGGAAATCACTATTTGTAGAAACTGTCAGGTC
ST6GALNAC6	0.999	.0319605	2.203746	-4.12	0.5010227	GTTACCTTCCTTGAAGCAGTTAATGTTATGAGATTTTGTGTCTCCTTTTAGACACAGTTA
AKAP12	0.999	.0104633	2.65564	-3.85	0.5003669	AGAAAGGAAAATGTCCTATCAGAAAGTGGTCCTCCATGTTTGCAGAAAACCCAGGACCCA
IGHV1-2	0.999	.002203	-3.218806	-3.49	-1.6538247	GCCCTGAAACTTCTGTGCATAGTTTGTGCCACCACTGTTAGGGTTGATCCATCCCATCCA
ORM2	0.999	.014338	-2.403793	-4	-1.2538799	ATTCTATAAATGCTCTCAGAAAACAAAAGTCCAAGAGGATTTCCTATCCCCAGGACCTGG
MESD	0.999	.004661	-2.9654	-3.66	-1.2253182	TGTTTCACAGTACAGGATCTGTACATAAAAGTTTCTTTCCTAAACCATTCACCAAGAGCC
AMN1	0.999	.033902	-2.436744	-3.98	-1.1305032	ACAAAACTCCGCATTAATGAGTTTATTGAAGATTCAGGTTAACCTGAATCAAGTTAACCC
TMEM18	0.999	.0297	-2.16542	-4.14	-1.0359545	CCCTATAGGCCAGAATTTTCCAATTAGAGGAATTCAGTTATATGATGGCCCCATCAACAT
EPHA2	0.999	.0297	-2.255366	-4.09	-1.0246753	GAAGCTTGTATTCCAAATGAAAATTTCCTACATCTGAGGCATCTGTGTAAAAGCTAGGAA
BMI1	0.999	.026752	-1.737827	-4.35	-1.023961	TCCTGCCCCATTATCTTGATCCGGTGCGCCATGTTGAATCCCCCTAACCGCTGCTTGAAA
TMEM150B	0.999	.025179	-1.649265	-4.39	-0.9533247	TTCATCTTGGGTAACGTCTACTTCTGGCTGCAGCTCCTCCTGTGGAGGCTGAAGAGGCTG
ANO7	0.999	.025179	-1.724295	-4.35	-0.9526981	AACTCCATTCCAGTGCAAGATTCTCATGGTTGAATTCATAGTGGGCTTCTATGACTTCTG
TTLL11	0.999	.03207	-1.544202	-4.43	-0.9288766	ATGCATTCTACCACTACATTTTGGTGCTATTTAAGGTGTGCAATTTTCTATAGGTGACTT
FAM169A	0.999	.1880487	-1.333772	-4.51	-0.8924675	GTGGAATGTGACGTCCCTGGAGACCCTGAAGGCTTTGCTTGAAGTCGACAAAGGGCACGA
FKTN	0.999	.0372943	-2.136908	-4.15	-0.8753961	CCTGTCCAAGGTTCCCTCTTGTCAGATCTGAGATTTCCTAGTTATGTCTGGGGCCCTCTG
RASL12	0.999	.0489122	-2.01627	-4.21	-0.8717597	AGAAAAGGAGACTTCGGCTACCCAGAGAAGTTCAGTGCCCAGCTCTACTGAGAAGAATGC
CENPT	0.999	.0561612	-1.95308	-4.25	-0.8503734	AGACCTGATTTATCATGTGCAATATCTCACACATCTGTCATTTCTATTCTACCGCAATTC
ZBTB34	0.999	.466393	-0.733716	-4.68	-0.8446201	AGTTTGCATCGCTGATCTTCAGTACCTTCACCTGTCTCAGTCTCTAGAGCCCTGAAAAAT
RASA1	0.999	.0668143	-1.871854	-4.29	-0.8349708	ACCAGTTCGATTCCATCTGTGGGTCTTAGGTTTCAGTTACCTGAAGTCAAATGTGGTCCA
RRP1B	0.999	.0247778	-2.311381	-4.06	-0.8318117	GTTCTGTTTTTGGAGGCTGGAAGAGCAGACAGCACATCCCTAAACTGGTTGCTGATTATA
TERT	0.999	.2514052	-1.15976	-4.57	-0.827039	TTAAATCAGTCAGTGGCTGGGCCCAAAGGCACTTTTGGGTAAGATAAGTGAAGAAATGCC
MTBP	0.999	.2196839	-1.24223	-4.54	-0.8051006	CAGACTGTAAATCTTGGAAAAGAAATCTGCCTGAAGTGTGAAATCTCTGAAAACATACCA
class V)(ADH6	0.999	.1417279	-1.491882	-4.45	-0.8006494	CCAGTATGAGAAGATGGCAGAGAAGAACCGCAAGGATGCCGAGGAATGGTTCTTCACCAA
SPDEF	0.999	.0719905	-1.836301	-4.3	-0.7941818	CAAGGGCTTGAGTGGATGGGATTGGTGTGCCCTAGTGATGGCAGCACAAGCTATGCACAG
UBL4A	0.999	.1038184	-1.655421	-4.38	-0.7807143	CAACAATGAAGTTAATGGATACCCTCTGCCTTTGGCTCAGAAATGTTATAGCAAAAATTT
ALG13	0.999	.1548083	-1.443589	-4.47	-0.7797662	ACATGTGCAGTCACTGGTGTCACCCTGGATAGGCAAGGGATAACTCTTCTAACACAAAAT
PHF13	0.999	.0912764	-1.720323	-4.36	-0.7689708	GGTAGTTTCCTCCCCGCAAGTAACAAAGGAGGACAAGCCCCAGCTTCTCTAAGATGCCTT
KRT24	0.999	.041137	-2.093771	-4.17	-0.7662078	TTCATCTTGGGTAACGTCTACTTCTGGCTGCAGCTCCTCCTGTGGAGGCTGAAGAGGCTG
MCM3	0.999	.1433287	-1.485788	-4.45	-0.7661786	CCATTCCAGTCCTTTTCAAATCCAAGACTGAAATGAATTTTTCTAGCTATTTATCCCATC
SLC35G3	0.999	.0785126	-1.794449	-4.32	-0.7624578	GACCTGATTTATCATGTGCAATATCTCACACATCTGTCATTTCTATTCTACCGCAATTCG
ITGB1BP1	0.999	.0035976	-3.048797	-3.6	-0.7573019	CAATGATCCTCAAACTGGATGATCGAATCCCGAATAATTGGGAATCAACAGTAAAGGCCA
TBX2	0.999	.0898235	-1.728309	-4.35	-0.7524026	CAGAGGCTCTTCAGTGATTTCTTGCTATTGACCGATGCTTCACTGTGCCAAAAGAGAAAA
MSRB2	0.999	.1683259	-1.396925	-4.49	-0.7497403	TAACACAGAAGCAACCAACTACAGTATAGCCTGATAACATGATTTCTTAGCTGACATTAA
BRMS1L	0.999	.1772763	-1.367599	-4.5	-0.7471851	TATGGGATCCACAGATATCCGGCTTTAAAATCCATAAACTTGAGCCCTAAAACAAACAAA
GABPB1	0.999	.3252479	-0.993055	-4.62	-0.7465357	GACGCCGATGACCTATGAAGTCAAATTTGACTTTACCTTTCCTCAAAGATATATTTAAAC
PKD1	0.999	.0049071	-2.937598	-3.68	-0.7434156	CCCTGCTGTTTGTGACATCTAAAAGAGATCTTACCTCCTTACCCTCAAAAAATTAAAATT
ZNF484	0.999	.389885	-0.86702	-4.65	-0.7376851	GTTCCAGAGGGTCTGATGTCTGGCACCTCAAGCATCAGTTTTTACTATATTATGATAAAA
ARF1	0.999	.1564257	-1.437843	-4.47	-0.729013	GGATCCTGCTACCAGGTTATGATAGATTTTATGGTATGTCTCAAGATATTGAGATAAAGG
mutated) 1(MUM1	0.999	.2054315	-1.28216	-4.53	-0.7277338	CTGGCCCAGCTTCGCTGCGAGATGGAGCAGCAGAACCAGGAATACAAAATCCTGCTGGAT
MYOM3	0.999	.2269635	-1.222571	-4.55	-0.7250065	AAGTTGAGGTATACATACCCCAGTTCAAATTAGAAGAGCATTATGAACTCAGATCCATTC
MMP19	0.999	.0356856	-2.156131	-4.14	-0.7180877	CATCAGATGCCTTCATCAGCTGGTATTTTGCCTAAGATCTATTTAAGATAACCTTTTCTT
CEMIP	0.999	.3825002	-0.880705	-4.65	-0.7078864	AAATTAATGGCAACTGCACAGGCATTAAAATCTACGTTTCTGATGATGGCAAAGCTCATT
CDC42BPB	0.999	.1778792	-1.365665	-4.5	-0.7056786	TAATCGAGGAGCTGCAGAACAAGATCCTCACAGCCACCGTGGACAATGCCAACATCCTGC
GRID1	0.999	.1666182	-1.402656	-4.49	-0.7055	GGGACCATTTCAGTAGAGCCTTTTAAGACACATGTTTTGGAATACAGTCAAATGCGTGCT
PLGLB2	0.999	.1280411	-1.546347	-4.43	-0.7036364	GCCCTTAATCCAAGTGTTGCCTATAAAATTATTTTCTGTTTTCTCACTCTAGTTCCCCCA
IL9	0.999	.1392874	-1.501278	-4.45	-0.7002045	TATATCAAAGAGCTGTGCAACCTCTTCGAGGCCCACAAACTTAAGTTCAACATCCCTGCT
SRSF12	0.999	.0636414	-1.894818	-4.27	-0.6992338	ATGAGCATTGCTTTCCTGGACCCAGGAAACATCGAGTCAGATCTTCAGGCTGGCGCCGTG
PKNOX1	0.999	.1989073	-1.301131	-4.52	-0.6968864	CCCAGCTTCTCTGCGAGATGGAGCAGCAGAACCAGGAGTACAAGATCCTCCTGGACATGA
SLCO3A1	0.999	.3312599	-0.980676	-4.62	-0.6967078	TCTCCAGAAAGCTAAAATTTAATTTCTTTTTTCCTCTGAGTTCTGTACTTCAACCAGCCT
SLC44A2	0.999	.2287521	-1.217812	-4.55	-0.6929026	TGTTTTCAAAAGTTTCCATGTCCCCGCCTGCAACCCCTGAGGTTGGGAAGTCCACGTCAA
INSIG2	0.999	.0942386	-1.704361	-4.36	-0.692461	GAAAATTGTGAGGGGAAAAATTATTTCTATTTGTACCCTGTTGTACCCAGCTTAGGTTTC
FABP6	0.999	.0792629	-1.789822	-4.32	-0.6905682	GGAGATTGAAAAAAGAAGACCTGATTTATCATGTGCAATATCTCACACATCTGTCATTTC
PCDHB11	0.999	.2227551	-1.233879	-4.55	-0.6904805	GACGTCATTTGAGAAAATGAAAGAAAATCCCATGACAAATCGTTCTACAGTTTCCAAATC
SV2B	0.999	.0289838	-2.245454	-4.09	-0.6866818	AGCTGGCTTGAGCAGTGGGTATTTTCATAAGGCATATATGGGCATCACAGAACACTTGGA
TMPRSS15	0.999	.022764	-2.346553	-4.04	-0.6816429	ATTGTATGACCTTCAGGCCTGGATCCACTCCTGTGTAGCACAGAGTTTTGAAAAGCACTA
NPHP1	0.999	.3156549	-1.013129	-4.62	-0.6784318	ATTGGCTGAGAAGTACCAAGACCACGAGGACATCATCATTGCTGAGCTGGATGCCACGGC
DPYSL4	0.999	.1620783	-1.418116	-4.48	-0.6781299	TTTGGAATCTGTATTTTTAACAAGCTGCCCAGTGAAAACCATTTCCTCCTCGTCGTGGCG
MYO9A	0.999	.0867768	-1.74541	-4.34	-0.67725	CAAATAGCATAAATTTCTCAAATCTGATTCCCCTGTAGCCATATAACCTTAGGTTCTTGC
ARPC5L	0.999	.0814797	-1.776367	-4.33	-0.6747175	CACTTGCAGTGGAGATGATTCCATTGTAAAATGTTTCTTCATTCCCAATAGCACAATATA
CSMD2	0.999	.0380875	-2.127696	-4.16	-0.6721981	GAAGTCCCATTAACTTAAAGTATATGTTTTCAAATTGCCATTGCTACTATTGCTTGTCGG
C16orf46	0.999	.1202066	-1.579651	-4.42	-0.6717208	TGATCCTGAAGATGGTGAAACAATATATGCACAGAAGTTCCAGGGCAGAGTCACCATGAC
PPP6C	0.999	.0948192	-1.701281	-4.36	-0.6695	AAACGTGATTAAAGAGATCACTTTGTCCGGATGAAGCAGCTGTTGGCATTATCCGCTGAG
SEMA3A	0.999	.0616584	-1.909669	-4.27	-0.668026	TTACTAGCTCTGCTATGCAGTGGGTGCGACAGGCTCGTGGACAACGCCTTGAGTGGATAG
TSN	0.999	.1404687	-1.496714	-4.45	-0.6660487	GACCAGATCAACCCTTCCATTCCATCGATTTGGACCGCAGTTTCGGGAATGATGATTTCG
C9orf69	0.999	.1080504	-1.634972	-4.39	-0.6653279	CCATGCACAAGGGCTTGAGTGGATGGGATTGGTGTGCCCTAGTGATGGCAGCACAAGCTA
DOCK9	0.999	.0182689	-2.436388	-3.99	-0.6645747	ATCTCCTTTCAGGGAATTTCAGGAACTAGAGATGACTGAGTCCTCGTAGCCATCTCTCTA
EFCAB1	0.999	.1375271	-1.508137	-4.45	-0.661513	TCCATCTCAATGTCCATTCCATTATTTTGTAGCTACAATAAAGAAACATGGGCAGCAGTG
TAC1	0.999	.0350832	-2.163525	-4.14	-0.6554545	CAGGACTGATCGATTTGCACCCACCTTTCTGCACATAAGTTATGGTTTTCCATCTTATCT
VSTM4	0.999	.0956955	-1.696662	-4.37	-0.6546494	GTAAGATTCTGTTCTTATCCGGCCACTGCCTGACCTTAGCTCTTCTTTCTCCAATGATAA
Hsp40) member C1(DNAJC1	0.999	.1826938	-1.350397	-4.51	-0.6524351	AGGATGCCACCATGACTAAAGAGAGTGCAGTGATCGCCAGGGATGGTAAAATCTACAAAA
NCEH1	0.999	.0659526	-1.877998	-4.28	-0.6521948	GCATCCAAGCATGATGAGCCCTCTCACGGTGCAATGGAGTGCACGGTCTGAATCTGCACA
HNRNPDL	0.999	.072298	-1.834257	-4.3	-0.6495779	TTCTATGTGGACCTGGAGAGGAAGGAGACTGTCTGGAAGTTGCCTCTGTTCCACAGACTT
ACOT11	0.999	.0106708	-2.648101	-3.86	-0.6466721	TGGCTAGAGCTTCTGTATTTTCAAAGACTGCCACGTGCCTTAGGAATACTGTTTTATCTC
LOC341378	0.999	.2098143	-1.269667	-4.54	-0.6463247	GCAGCGGCCTGCTGAGATCAAAGACTACAGTCCCTACTTCAAGACCATCGAGGATCTGAG
CEMIP	0.999	.3270104	-0.98941	-4.62	-0.6460909	GTTCACCTTCTATTTTGAAGACCCGCTTCTTCCTCAGGTACCTGATCTTGAAAACGACCT
FCAR	0.999	.0577019	-1.940559	-4.25	-0.6429253	AAGGAATATTTATGAAGTCTCTGCAACTTGCCAGGTGCTTTCACTATGCAACCCTCCCTG
HRASLS	0.999	.02166	-2.367034	-4.02	-0.6424351	GTGGTTGGTGTCTCTGCCATCTACATCTGCTGGAAACAGAAGGCCTGACTGACCCTCAGT
STK38L	0.999	.2419139	-1.183602	-4.56	-0.637211	TTATTTTCTCTCAATTCCCTACTGCCTGTTTCTTACTTTGAACCTGGAGGCAGCCTGCAG
NUDT15	0.999	.1383719	-1.504836	-4.45	-0.63625	AAGGTAGTGGAAGCTACAACAAGGGGAGCGGAGTTCCTGATCGCTTCTCAGGCTCCAGCT
MAP4K5	0.999	.0024998	-3.176411	-3.52	-0.6342922	GCCTTCATTCTGGAGGTATTTGAGTTTGCATCTCCTGATAAAGAATAAATCTCAAAATTG
GNAI1	0.999	.1447156	-1.480552	-4.46	-0.6341461	CCAGGTGGAGGATGAAGCTGACTACTACTGTTACTCAACAGACAGCAGTGGTAATCATAG
HAUS6	0.999	.4789144	-0.713156	-4.69	-0.6232273	ATCTCCCGGGCTGGCCACCTCCTTGACCAGCATATCTGTTTTCTGATTGCGCTCTTCACA
SMIM11A	0.999	.0565285	-1.950068	-4.25	-0.6216169	ATGAAAAGCTTTCCTGCTTGGCTCTTATTCTTCCACAAGAGAGGACTTTCTCAGGCCCTG
XAGE2	0.999	.2717266	-1.1108	-4.59	-0.62125	TGTTGAGAAGCTGCTCACACCAAGGGGCTGAAGTAGGAATCACCTGGGAAACAGGGGGCA
STARD13	0.999	.1557628	-1.440192	-4.47	-0.617961	TTTAAGACACATGTTTTGGAATACAGTCAAATGCGTGCTGCTGTATGACCAGCCTATTTG
KHNYN	0.999	.16249	-1.4167	-4.48	-0.6163929	TGGCGTGGACCTGAGCTGCATCCTGAATGAGATGCGCAACCAGTACGAGCAGATGGCAAA
SLC12A4	0.999	.2083177	-1.273911	-4.53	-0.6159448	CCAGGGTTGATTCCAGTCAATTCAGCAGGCCTAGATTTATTTTCCTTAATAATAATTAAG
KDM1B	0.999	.1497802	-1.461759	-4.46	-0.6122857	CAGTGTAGTTCCCAATATGGCCTCATACTAACTTCAGATACCCCAGTGTATCTGAAGAAA
AGMAT	0.999	.0438007	-2.065888	-4.19	-0.6075649	AGACCGAAAGATAAGTCCACAGTCAAAAGAGAGTGAGTTACGTATCTTGGATGAGATCCC
SPIB	0.999	.2405971	-1.186963	-4.56	-0.6075097	AAGCAGTAAGGTGCTGCAAAGGACCCTTCTTTTTGGCCATAAAAACCTGAGAAACTGATT
MAP3K10	0.999	.4723433	-0.723907	-4.69	-0.6064286	CCAGTACACAACACGCATCTCGCCAGTTTGCCTGGCATCCTCAAACGAGGCTCTGACTGA
ZBTB14	0.999	.0483824	-2.021199	-4.21	-0.606224	GATGTGAGTGTGTTTCATCAAACATAGCTCAGTCCTGATTATTTAATTGGAATATGATGG
ANTXR2	0.999	.0662139	-1.876128	-4.28	-0.6039318	ACATATCATTGTGACCCCAGAAGATGGAATCTATGGTTGGATCTTCACCAGGGAGAGCAT
APOM	0.999	.004762	-2.948453	-3.67	-0.602763	TAACAAGAGTTATTTTTATGTAAGCTTCTCTCATTCCTCCACTGTGCGTGCTCGGGGGCT
NUDT16L1	0.999	.063874	-1.893102	-4.28	-0.601276	TACCTTCTCTGGATAATGAGTATGGATGACATACCTTCTAGTGTCTGCTGTCGCGCTTAC
GLDC	0.999	.0711147	-1.842161	-4.3	-0.5988312	GACATACGCTCCATTAGCATTCCGATATTTCAGAGAACTTTTTGGTATCAAGCCTGATGA
RGS1	0.999	.1721431	-1.384275	-4.49	-0.5986266	CTCTGGCACACCATGCAGAACCTGGAGATTGAGCTGCAGTCCCAGCTCAGCATGTTAGGA
AGT	0.999	.0084675	-2.73613	-3.8	-0.5986201	ATTATTAAAAGATGCATTTCCACTTTGACAGTGATTTCCCCACCCGAGAGCCTGGTTGGG
HDGFRP3	0.999	.2229059	-1.233471	-4.55	-0.5983896	ATCTGCAGAAACCAGGGAAATCCCCTAAGCTCTTCCTCTATGATGCAAAAGATTTGCACC
PARD3	0.999	.1214749	-1.574143	-4.42	-0.5967208	TACGGACTAGCAATATCCATACCCGCTACTTATTTTGTAAATCTCCTTTCTAAAGTTAAC
ZNF304	0.999	.1689243	-1.394927	-4.49	-0.5958019	AACCGAAAAATCACATTTTTCTTGATTTCAAATATGTTCTACGGCCTTACTGTTGGGATG
TRPC1	0.999	.2810425	-1.089219	-4.59	-0.5944383	GTGCTGGCCCTGAGTACTGAACTTTCTGAGTAACAATGAGACACGTTACAGAACCTATGT
DIDO1	0.999	.0410313	-2.09491	-4.17	-0.5938279	CAAGCATGATGAGCCTTCTCATGCAGTGGAATGGAGAGCATGGTCTGAATCTGCACAGAG
KCTD10	0.999	.2713675	-1.111643	-4.59	-0.5886851	GCACTGGCAATTCCAAACAATGTCAGTGTTAAAATGCTTCTCCCTGAAAAAGAGAAAAAA
EREG	0.999	.2404034	-1.187458	-4.56	-0.5878636	TGTCTAAGTCACAAATCTGAAGAAATAAGAGATTGTCTGTAGTTGATTGAAACGAGGGCA
BRD3	0.999	.1854251	-1.341871	-4.51	-0.5866136	ATCAAAATGAAAGATGCAGAAATGCAGCAACTATTGCAGGGATTCCGTGAGACCAAGGAC
ACSS3	0.999	.2178514	-1.247254	-4.54	-0.5854545	TAGATTCTTATAATATTATTTAAATGACTGCATTTTTAAATACAAGGCTTTATATTTTTA
PHF2	0.999	.0127466	-2.579257	-3.9	-0.5831169	TGTTTGCCTGTCACTGGGCTCAAGGAATTACAAAGTATAAAGAGTTACAGTAGCAATTCC
POR	0.999	.1756417	-1.372869	-4.5	-0.5768669	TGCAATCTGGGTCTGAGTTGAAGAAGCCTGGGGCCTCAGTGAAGGTCTCCTGCAAGGCTT
C7orf49	0.999	.0774749	-1.800907	-4.32	-0.5764481	CTGAATCCTGTGTGATCTTCTCACCTGATCTAATAAGAATAGTGACTGAGTTCAGACCGG
SLC9A8	0.999	.0652942	-1.882738	-4.28	-0.5753377	GGCACAAGTGGACTCTGCTATCCCATTCGATCCATTCGTTGTCCGTGTCAAAAATCAATT
NF2	0.999	.1107866	-1.622096	-4.4	-0.5743571	GAGTTTGACACGAATATGGATGCAGTACAGATGGTGATTACAGAAGCCCAGAAGGTTGAT
PCDHB10	0.999	.266723	-1.122608	-4.58	-0.5712792	CAGTAGCCCTGAATGAAAAATTTGATAAACATTATGAGCAGCAAATGAAGGAATCTACAC
FZD8	0.999	.3964282	-0.85503	-4.66	-0.570539	GCTCTGGTGGACATATGTCTGCAGCCTAGTGTTCAAGGCGAGCGTGGACATCGCCTTTCT
MSR1	0.999	.0093071	-2.700348	-3.83	-0.5696461	TTTGAGTGCTTCTGAATACATGACTGTGCTCTGAATACAGAGCTACCACTGCGGGGTAAA
PML	0.999	.0517339	-1.990772	-4.23	-0.5691104	GTAATAAATTTTATTGTTGTTTTAAGAAGCGCACCAACGGTCCAGGTTTGGAGTGTACCA
RFX1	0.999	.1567848	-1.436573	-4.47	-0.5685682	TTGTAGATGAACTCTTCTCAACTCTGTTTTGCTATGCTATAATTCCGAAACATACAAGAC
LGALS9	0.999	.2727438	-1.108419	-4.59	-0.568487	TTCTTCCCCTGTTGAAAAGCTTTCTCATGATCATATTTCACCCACATCTCACCTTGAAGA
HIST1H4G	0.999	.2027095	-1.29002	-4.53	-0.5678214	GTGGCCAGCTGACAATATACATCTCTGTATCTCCTATTTTTATCTTTATGCTAAATGCAA
HOXD11	0.999	.3377943	-0.96739	-4.63	-0.5675065	AGACGCTCACGCTGGCCAAGAACTACATCAAATCGCTGACGGCCACCATCCTGACCATGT
KLF12	0.999	.3974619	-0.853147	-4.66	-0.5652208	GTTCACCTTCTATTTTGAAGACCCGCTTCTTCCTCAGGTACCTGATCTTGAAAACGACCT
RCE1	0.999	.2268732	-1.222813	-4.55	-0.5646494	TCTTACTCACCTTCCTTTAGTCTCGTCCTGTGGTGTTGAAGGGAAATCAGCCAGTTGTAA
PPP1R36	0.999	.1957106	-1.310598	-4.52	-0.5598019	GGGAGATAGTGTACCCCTTCCAGGGGGACTCCACGGTGACCAAGTCCTGTGCCAGCAAGT
SRSF10	0.999	.1885436	-1.332253	-4.51	-0.5585649	CATACTGCCTTGTTTAATGGTAGTTTTACAGTGTTTCTGGCTTAGAACAAAGGGGCTTAA
DOK2	0.999	.2908231	-1.067092	-4.6	-0.555724	CCAGCCAGTCCAGCTGAATGAAACCTGGGTCAACAGCCATGTGGACAACTGCACCGTGTA
GPR107	0.999	.043168	-2.072374	-4.19	-0.5520162	TCTTGGAATTCAATGACCTAATTCTGTGTATGGTTGCAGTGTGGAGATTGCCTCTTGTCT
OR4A15	0.999	.0625607	-1.902862	-4.27	-0.5513831	CACCTCCTCATTGCCAAAGGACCTGGCTTAGATACAAGTAAAAATATATGATTAAATAAC
PHF14	0.999	.4156294	-0.820535	-4.66	-0.5496364	GGCCTCTCCTCAGGCCAGAAGTCCAGCTGCTGACGGGCAGGTCCCGTTTCTCCAGAGTTG
MAPKAPK3	0.999	.254107	-1.153092	-4.57	-0.5496331	TCCTGCAGCAGATCAAAATGGTAGCCCCTTTTCTTTCCATGAAAAGAATTTCTACGGAGA
Hsp70) member 6(HSPA6	0.999	.0407555	-2.097892	-4.17	-0.549474	GTTTGGTAGTATTTCTCGCCATGCTTTGCTCATGCGCAATGAGACTACAACTAGGGTGTA
LRFN2	0.999	.1250676	-1.558789	-4.43	-0.5486981	GGATGCCAGTATTCTCATCCCCTCACAAATAAAGAGCCTTCAAGCTCTTGCAGTCAACAA
NCF2	0.999	.1553514	-1.441655	-4.47	-0.5471981	TGGCTCTCCTATGAAATAATGCTCATAAACATCCCTTGTGAGAAGGATCAGATCAACATA
HEXIM1	0.999	.1653936	-1.406794	-4.49	-0.5462565	GCCACTCAGCTACCTAATTCCTCAATGACCTTTATCTAAAATCTCCATGGAAGCAATAAA
TDRD5	0.999	.1794598	-1.360618	-4.5	-0.5433506	ACCCTGTTTATACTCAAGTTGTCAAAGAATTCCAGAGGGCATATGAAGCCATTGTTAAAA
ERP44	0.999	.1224277	-1.570036	-4.42	-0.5430779	CCAACAATATTTGAAACCCTTATCTGCGTGTACAAGTTTGCTACTGGTTTTGTTTTGCTC
SFXN2	0.999	.2897108	-1.069582	-4.6	-0.5427532	CACATATCGAATTACCAACCACTCACACTTCCGGATGGAGGGAGAGGTTGTGCTGACCAC
IL18	0.999	.0149217	-2.517315	-3.94	-0.5401948	TCCCACCCTGAGGTCTACAGGGTCCTGTTTGACTACCAGCCTGAGGCCCCAGACGAGTTG
ATIC	0.999	.1976024	-1.304981	-4.52	-0.5399383	ATACCTGCAGATCAGCAGCCTAAAGGCTGAGGACATGGCCATGTATTACTGTGCGAGATA
PLEKHF2	0.999	.0744446	-1.820192	-4.31	-0.5394351	TGTACCCTGCAGAATCTACCAAGGCACTCTGTAGCAAAGCAGGCCTTCCACCTGTCTCGG
GID8	0.999	.1609231	-1.422104	-4.48	-0.5390292	GCTCGTTATCAAGTGCTGTTTTTACACAGTAAAACATGGGCAAAATTACTCTTCTTTAAG
SNRPA	0.999	.0959135	-1.695519	-4.37	-0.5381916	TGGGGTAAACCACAGTCTACATAGGGAAGGACTCTTTCCTTAGCCTTCTCTTATTGATTG
DLST	0.999	.2061836	-1.280002	-4.53	-0.5372565	GCTGATACTGCATTCCATTCATTCATTGTCTGAATTCAAGTTTGTGTTTTTCATGAAGGC
GMFB	0.999	.1846781	-1.344194	-4.51	-0.5366916	TTTCAATGGTAACACCAACTACGCACAGAAATTCCAGGACAGAGTCACCATTACCAGGGA
TRIM6	0.999	.1569212	-1.436091	-4.47	-0.5361526	CTTTCTTGTATTTCTCTCACGTTAACAAAATTGGTTCAGCATCTACCATGGGCTACATGC
DHRS2	0.999	.1213425	-1.574716	-4.42	-0.5350455	TTCTCCGGCCACAACCTTGAATACTTGCTGCGTCTGGCAGACTTCATGCAGCTCTGTGGG
ANO1	0.999	.1321022	-1.529721	-4.44	-0.5339968	TTACACATGCGTGTTGTGGACAACTTGAGAAAACACTTGTCTGCAGGAAGATGAAATTAA
PTPN12	0.999	.0353829	-2.159833	-4.14	-0.5328344	ACAGATTTAACCCAAAGGACATAGTTCAACTCCTGGTCTTGACAGAGGAGAAGCCCTCAA
OMP	0.999	.2836462	-1.083277	-4.6	-0.5321169	CACATCCTGGTCCCTAGCAAGGTATAGATAGCCTCTGTGTCTTAGGATACCCCGGGTGCT
PIGH	0.999	.1890336	-1.330753	-4.51	-0.5312857	AGATGGTGAAACAATATACGCAGAGAAGTTCCAGGGCAGAGTCACCATAACCGCGGACAC
SETX	0.999	.1078542	-1.635905	-4.39	-0.5304318	TCAGGTGACCCTGATCACCCTCTGGGTGTACGTGTACGTGGACCCAAACAATGTGCTGGA
RPS3A	0.999	.4097511	-0.830989	-4.66	-0.529737	CTGAAGAGAGACTTAAGATGAAAGCAAATGATTCAGCTCCCTTATACCCCCATTAAATTC
TRAPPC13	0.999	.0941151	-1.705018	-4.36	-0.5282727	GACTTGGTGCCACCGCCAGCAATAATTGACAAACTTTTCCTGGACACTTTACCTTTCTAA
PRR13	0.999	.1423734	-1.489418	-4.45	-0.5271526	GCGGATGAATGGTACTTTCCACAAGTGCATTTGAGTAGAAGCATAACCTATTCTCAGTTA
PILRA	0.999	.2290008	-1.217153	-4.55	-0.5267825	TTCCGTTCATTCTTTCAGTAAATGGTTGCAGCACATGTTTTACATGTCAGGCAGTGAAAC
TXNRD3	0.999	.2998995	-1.047015	-4.61	-0.5266753	GCTATCAGAATATTGACTATTAGTGCTTTCATCACGCGGCTCCATCCAAACCCCTGCTTT
PCDHAC2	0.999	.2051256	-1.283039	-4.53	-0.5258799	TGGCATGTCATTTGACCCAATGGCTTTTTTAGTCATTTATCTTCCTAACACCTATGAGAG
CRIP1	0.999	.0266005	-2.281676	-4.07	-0.5255552	GCTGTGGAGACCCGGAAGAGTTAGATATAATGTCATTTGTTGTAATTCAGTTTCATAAAA
AMD1	0.999	.1070915	-1.639547	-4.39	-0.5234156	TCATATACATTAAGTTGAGCCATATGTAATCACTGTGTTTGTAGGTTAGAAACAGCTGAG
ZNF655	0.999	.0483165	-2.021815	-4.21	-0.5227922	GGAAGTTGGGTGATTTTAAGTTATTGTTGCCAAAGAGATGTAAAGTTTATTGTTGCTTCG
C3orf22	0.999	.0639571	-1.89249	-4.28	-0.5225617	CCCTCCCTGGGAGAAGCTGCAGCTCTGAGGCTACGGTCTTGCAGGTCAGCCACCACCTGG
KIF7	0.999	.0371003	-2.139187	-4.15	-0.5217143	AACCGTCTGAGTCTTGTGCTCTTCAAGACAAAACAGATTGCGTCGCTGACAAGTTCTCAA
HTR1B	0.999	.3775627	-0.889948	-4.65	-0.5208831	AGCCCTGTTAAATGGTCGTGGCCAATTATGTCATAGAAACTGTATGAACAACCAGATTTA
ALG9	0.999	.261752	-1.134494	-4.58	-0.5206006	AGCAACACCGAGGATTTGCCTCTGGTCACCAAGATGTGCCACATAGGCTGCCCCGATATC
PHF19	0.999	.0699512	-1.850042	-4.3	-0.5201948	CCTGGCTCATTTCTTGTTTCGGCAAGTCTGCTAAAAGATTCACTCAGCACAGCAGGGAAA
ZNF416	0.999	.1164463	-1.59626	-4.41	-0.5194935	TCTCTAAGGGATCTCTGTTGCTTGGAGAATAAACCCTCGGATTCCTTCCTTGGCTCTCGG
RNF112	0.999	.0175159	-2.45335	-3.98	-0.518763	CTTCTGGCAATTAAAGTTAGTCATGTTAGAACACTGTCTAGGAATGGTTGGAAAATCATA
TMEM43	0.999	.1041237	-1.653923	-4.39	-0.5185617	TCAAATCAGTTACATTTTCAGAAGAGACTCTTAGTTTAAAGCAGTGCCCCTCTTTAAGAG
AATK	0.999	.1086034	-1.632348	-4.39	-0.5182273	ACTCGGCTATTCCACTTCGATGATTCTCTGTCACAGGTGTCTTAAATACATGAACAAAGG
AKT3	0.999	.1665018	-1.403048	-4.49	-0.5177403	CAAAAGCTTTCTGGCTCTGAATCTATGGAAAGTGTGGATCATACTTCTGACTGCCCCATG
CXCL3	0.999	.3998663	-0.848779	-4.66	-0.5142955	GACAATTCCTTCTGATGCACAAGAATGAAGCCGAGTTTCAGAGGTGGCTCATTTGCTGTT
RBM41	0.999	.3838784	-0.878138	-4.65	-0.513961	GGAAGAATCTACCACTTTCCAGAGCTGGCCAAGCTCAAGTGACACAACACCTTCACCTCC
ELMOD2	0.999	.2697094	-1.115542	-4.59	-0.5123734	CTGGAAAAATTTATCAAGTTCCTAAACCGCAATGCATACATCATGATCGCCATCTACGGG
Knops blood group)(CR1	0.999	.1607258	-1.422787	-4.48	-0.5113149	TGAAAATAACACTAGGGCATAACAATTACTTCAGGGGATACGCCATCCACCCTGGCTCTT
CA13	0.999	.3753443	-0.894125	-4.65	-0.5109935	CTCCTCAATCTGGTTTATGAAACAACTGACAAACACCTTTCTCCTGATGGCCAGTATGTC
MORC2	0.999	.0140218	-2.541877	-3.92	-0.5084188	ACGAAAGATTTCAGTGTACCATGCGATACTGTTTGATAAGCATTTTACCCATAGTAGAAC
SMIM10	0.999	.1153218	-1.601311	-4.41	-0.5081266	TGTAAAGTGACTTGTCTAAAGTCACACAGATGTGAGTCATGCAGGACTTTGGGACTGCAG
RHBDD2	0.999	.0064375	-2.838377	-3.74	-0.507987	TTAATCATTGCTGCAAAATCTCACGTCCAGGAAGAATTAAACCCATCGCCTTGGGGGCAA

DEGs, differentially expressed genes; PDAC, pancreatic ductal adenocarcinoma; PNI, perineural invasion; FC, fold change.
